# Supplementary figures and images for: The Role of PnTCP2 in the Lobed Leaf Formation of Phoebe neurantha var. lobophylla
Source: Int J Mol Sci. 2022 Oct 31;23(21):13296. doi: 10.3390/ijms232113296 (PMC9653974; doi:10.3390/ijms232113296)

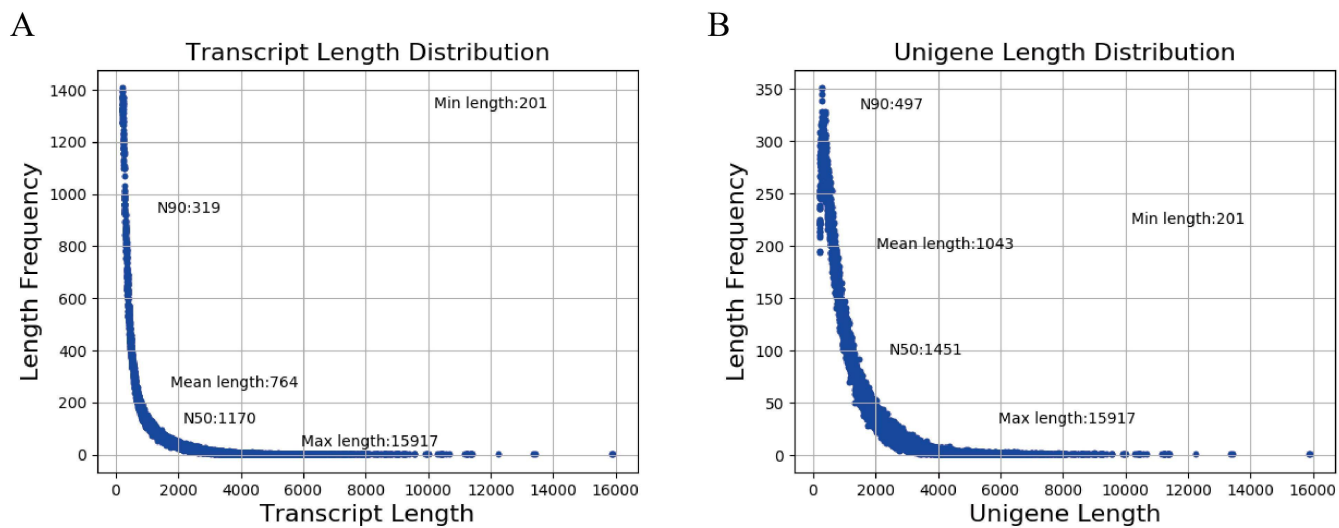

Supplement: Supplementary file 1 [file ijms-23-13296-s001.zip › Figure S1 Length distribution statistics of transcripts (A) and unigenes (B).pdf]
